# Circulating calprotectin levels four months after severe and non-severe COVID-19

**DOI:** 10.1186/s12879-023-08653-7

**Published:** 2023-10-03

**Authors:** N. Abu Hussein, C. Machahua, SC. Ruchti, MP. Horn, L. Piquilloud, M. Prella, TK. Geiser, C. von Garnier, M. Funke-Chambour

**Affiliations:** 1 Department of Pulmonary Medicine, Bern University Hospital, Inselspital, University of Bern, Bern, Switzerland; 2 Department for BioMedical Research DBMR, Inselspital, Bern University Hospital, University of Bern, Bern, Switzerland; 3 Faculty of Medicine, University of Bern, Bern, Switzerland; 4 Department of Clinical Chemistry, Bern University Hospital, Inselspital, University of Bern, Bern, Switzerland; 5 Division of Pulmonary Medicine, Lausanne University Hospital (CHUV), University of Lausanne, Lausanne, Switzerland; 6 Department of Diagnostic Laboratory Medicine, Inselspital, Bern University Hospital, University of Bern, Bern, Switzerland

**Keywords:** Long COVID, Calprotectin, Post COVID sequelae, ARDS sequelae, Post-infectious inflammation

## Abstract

**Background:**

Calprotectin is an inflammatory marker mainly released by activated neutrophils that is increased in acute severe COVID-19. After initial recovery, some patients have persistent respiratory impairment with reduced diffusion capacity of the lungs for carbon monoxide (DLCO) months after infection. Underlying causes of this persistent impairment are unclear. We aimed to investigate the correlation between circulating calprotectin, persistent lung functional impairment and intensive care unit (ICU) stay after COVID-19 in two university hospital centres in Switzerland.

**Methods:**

Calprotectin levels were measured in serum from 124 patients (50% male) from the Bern cohort (post-ICU and non-ICU patients) and 68 (76% male) from the Lausanne cohort (only post-ICU patients) four months after COVID-19. Calprotectin was correlated with clinical parameters. Multivariate linear regression (MLR) was performed to evaluate the independent association of calprotectin in different models.

**Results:**

Overall, we found that post-ICU patients, compared to non-ICU, were significantly older (age 59.4 ± 13.6 (Bern), 60.5 ± 12.0 (Lausanne) vs. 48.8 ± 13.4 years) and more obese (BMI 28.6 ± 4.5 and 29.1 ± 5.3 vs. 25.2 ± 6.0 kg/m2, respectively). 48% of patients from Lausanne and 44% of the post-ICU Bern cohort had arterial hypertension as a pre-existing comorbidity vs. only 10% in non-ICU patients. Four months after COVID-19 infection, DLCO was lower in post-ICU patients (75.96 ± 19.05% predicted Bern, 71.11 ± 18.50% Lausanne) compared to non-ICU (97.79 ± 21.70% predicted, p < 0.01). The post-ICU cohort in Lausanne had similar calprotectin levels when compared to the cohort in Bern (Bern 2.74 ± 1.15 µg/ml, Lausanne 2.49 ± 1.13 µg/ml vs. non-ICU 1.86 ± 1.02 µg/ml; p-value < 0.01). Calprotectin correlated negatively with DLCO (r= -0.290, p < 0.001) and the forced vital capacity (FVC) (r= -0.311, p < 0.001).

**Conclusions:**

Serum calprotectin is elevated in post-ICU patients in two independent cohorts and higher compared to non-ICU patients four months after COVID-19. In addition, there is a negative correlation between calprotectin levels and DLCO or FVC. The relationship between inflammation and lung functional impairment needs further investigations.

**Trial registration:**

NCT04581135.

**Supplementary Information:**

The online version contains supplementary material available at 10.1186/s12879-023-08653-7.

## Background

After acute COVID-19 disease various symptoms can persist. Previous coronaviruses, such as SARS and MERS induced persistent lung impairment after acute infection as well as signs of pulmonary fibrosis [1–4]. While severe fibrosis after COVID-19 is rare [5], lung functional impairment is observed after 3 months and later [6–9].

Long COVID is complex and the underlying mechanisms remain unclear. Clinical appearance is heterogenous with a wide range of possible symptoms affecting different organ systems. The most commonly reported symptoms include dyspnea, fatigue and cognitive impairment [10–13]. In addition, long COVID can occur regardless of the initial disease severity. There is a great clinical variety of affected patients [14]. A comprehensive review recently investigated the existing literature on the pathophysiology of long COVID and concluded that despite a large number of existing hypotheses to explain persisting symptoms after acute COVID-19, the mechanisms underlying long COVID remain unclear [15].

Ongoing inflammation is among the most debated hypotheses [15]. The inflammation-hypothesis is supported by research on biomarkers [16]. Different pro-inflammatory biomarkers have been associated with long COVID. For example, C-reactive protein (CRP), interleukin 6 (IL-6), and tumor necrosis factor alpha (TNFα) were significantly higher in patients with persistent symptoms after COVID-19 compared to patients that fully recovered [16, 17].

A largely unexplored inflammatory biomarker in the context of long COVID is calprotectin that correlated with disease severity in acute COVID-19 and may constitute a promising biomarker to predict the risk for a severe disease course, intensive care unit (ICU) admission, intubation, and mortality [18–22]. At present, its levels and relevance in long COVID are undetermined.

Calprotectin is the stable heterodimeric complex of S100A8 and S100A9, which are members of the Ca^2+^-binding S100 protein family, and it plays crucial roles in multiple biological functions, most importantly in inflammatory processes [23]. The main source of S100A8/A9 are neutrophils and monocytes, but expression can also be found in macrophages, platelets, or cells at the site of inflammation like epithelial cells [23, 24]. Once released, S100A8/A9 acts as an inflammatory modulator with predominantly pro-inflammatory properties, although anti-inflammatory properties are also known [25]. Its major functions include the recruitment of neutrophils and the induction of pro-inflammatory cytokines such as IL-1β, IL-6, IL-8, and TNF-α [25].

Fecal calprotectin is measured in clinical routine as a marker for inflammatory bowel disease activity. Moreover, we have previously observed that patients with idiopathic pulmonary fibrosis (IPF) have increased serum calprotectin levels compared to healthy individuals, indicating its involvement in fibrotic interstitial lung disease [26].

Our study aimed to investigate levels of calprotectin in patients after severe or non-severe COVID-19 with or without pulmonary impairment.

## Methods

### Swiss COVID-19 lung study cohort

The Swiss COVID-19 lung cohort is a prospective observational cohort study to investigate pulmonary and extrapulmonary effects following COVID-19 (Clinical Trial identifier NCT04581135). For this sub-analysis patients recruited in Bern and Lausanne were included.

### Clinical assessment

124 patients were recruited at the University Hospital in Bern (Inselspital, Bern, Switzerland). They were classified according to the severity of the disease into post-ICU, if they had the severe disease condition and stayed in ICU (with or without mechanical ventilation); and non-ICU, if they had mild or moderate COVID-19. To validate our results, 68 patients were included from the University Hospital in Lausanne (CHUV, Lausanne, Switzerland). This validation cohort consisted only of patients who were admitted to the ICU because of very severe COVID-19 lung disease. Diagnosis of COVID-19, disease management and routine follow-up were performed according to national recommendations [27]. Three months after the acute illness, the patients’ history, clinical data, laboratory, and lung functional tests were recorded. All participants were > 18 years old and signed a written informed consent before inclusion in both centres, which was approved by the local Ethical committee (KEK 2020–00799).

### Biobank/ serum samples

Blood samples were obtained from all participants at the first visit four months after the acute phase of the disease. Serum was isolated and stored at -80 °C. For the calprotectin analysis all serum samples were transferred to the Department centre of laboratory medicine in the Inselspital (Bern).

### Calprotectin measurement

All samples were analysed in the centre of laboratory medicine in Bern. A commercially available enzyme-linked chemiluminescent assay (CLIA) kit for circulating calprotectin was used (701,365, QuantaFlash, INOVA Diagnostics, San Diego, CA) according to the manufacturer’s instructions.

### Statistical analysis

Data is shown as mean ± SD. Quantitative groups’ features and calprotectin levels were compared by ANOVA test for parametric variables (with Tukey HSD as post-hoc test) or by Kruskal-Wallis Test for non-parametric (with Pairwise Comparisons as post-hoc test, p-value adjusted by Bonferroni correction for multiple tests). To determine an association between categorical data, Chi-Square Test of Association was performed. Moreover, Spearman’s correlation (r) was performed to associate the serum calprotectin levels and the lung function parameters. In addition, a multiple linear regression (MLR) was also assessed to determine the statistical implication of the patient’s features to the calprotectin measurement, considering calprotectin as dependent variable and age, sex, BMI, lung function and comorbidities as explanatory variables. Dataset and statistical analysis were performed in IBM® SPSS® Statistics v28.0.1 (IBM, USA). In all cases, statistical significance was assumed when p-value < 0.05.

## Results

**Fig. 1:**
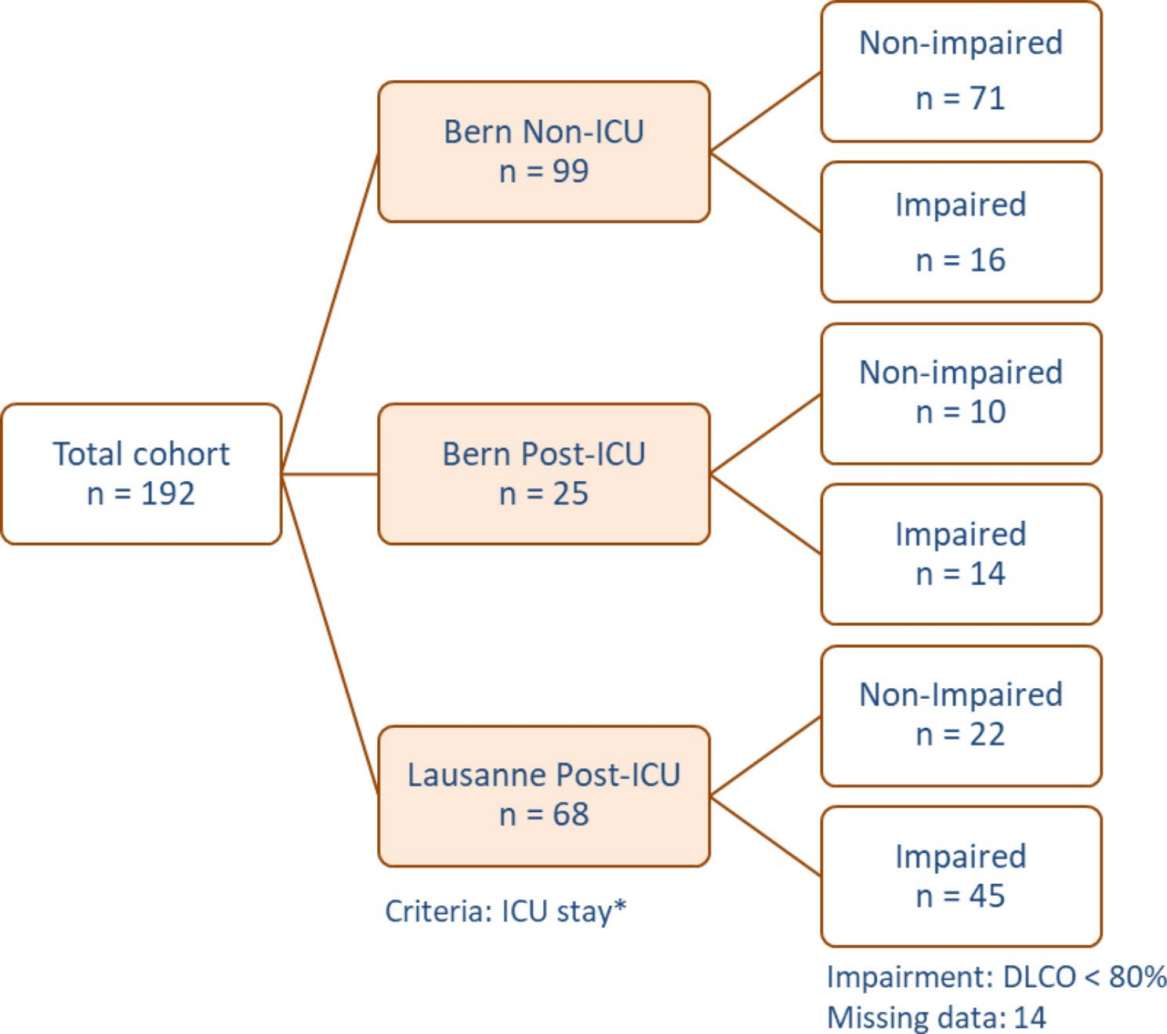
Post-COVID-19 Flow-Chart: Bernese and Lausanne Cohorts The flow chart shows patients’ group selection according to their ICU stay during the initial illness and impairment defined as diffusion capacity above or below 80% during lung functional measurement after 3 months

To assess the relationship of serum calprotectin levels and persistent pulmonary function impairment after COVID-19, blood samples from a total of 192 post-COVID-19 patients were investigated in this study. As illustrated in Fig. 1, participants were divided into three groups, which were recruited at the two centres in Bern and Lausanne. 99 non-ICU patients and 25 post-ICU patients from Bern were compared to 68 post-ICU patients from Lausanne. Some of the clinical data from patients have been included in a previous publication about lung functional impairment 4 months after COVID-19 [6].

### Post-ICU patients show greater lung impairment 4-months after COVID-19

Clinical characteristics of all three groups are summarized in Tables 1 and 2. Overall, mild-moderate COVID-19 patients from Bern (Bern non-ICU, n = 99) had equal numbers of males (44.4%) and females, with an average age of 48.84 (± 13.40) years and a close to normal BMI of 25.24 (± 5.94) kg/m^2^. In contrast, patients in the post-ICU groups were predominantly males (72.0% and 76.5%, in Bern and Lausanne, respectively) compared with non-ICU (p < 0.0001). These post-ICU groups were significantly older (Bern = 59.35 yrs, Lausanne = 60.52 yrs, p < 0.0001), and had a significantly higher weight (Bern BMI 28.65 kg/m^2^, Lausanne BMI 29.19 kg/m^2^, p < 0.001) compared to the non-ICU patients. Thus, the two post-ICU groups fulfilled the described risk factors for a severe course of COVID-19 such as male sex, advanced age, and increased BMI.

**Table 1:** Bern and Lausanne Cohorts´ Baseline Characteristics

	Bern Non-ICU (n = 99)	Bern Post-ICU (n = 25)	Lausanne Post-ICU (n = 68)	p-value
**Age, yr.**	48.84 ± 13.40	59.35 ± 13.60	60.52 ± 12.02	**< 0.0001** ^**a,b**^
**Sex, M (%)**	44 (44.4%)	18 (72.0%)	52 (76.5%)	**< 0.0001** ^**a,b**^
**BMI, kg/m** ^**2**^	25.24 ± 5.94	28.65 ± 4.55	29.19 ± 5.33	**< 0.001** ^**a,b**^
**Lung impairment, (%)**	16 (18.4%)	14 (58.3%)	45 (67.2%)	**< 0.0001** ^**a,b**^
**DLCO, % pred.**	97.79 ± 21.70	75.96 ± 19.05	71.11 ± 18.50	**< 0.0001** ^**a,b**^
**FVC, % pred.**	97.40 ± 15.42	87.65 ± 14.11	87.11 ± 20.96	**< 0.012** ^**a,b**^
**TLC, % pred.**	100.98 ± 17.79	86.70 ± 15.07	82.91 ± 18.85	**0.001** ^**a,b**^
**Desaturation index, %**	3.84 ± 5.19	5.17 ± 3.80	6.86 ± 3.93	**< 0.039** ^**a,b**^

^a^Bern Non-ICU vs. Bern Post ICU

^b^Bern Non-ICU vs. Lausanne Post-ICU

^c^Bern Post-ICU vs. Lausanne Post-ICU

Abbreviation: BMI: Body mass index; DLCO: diffusing capacity for carbon monoxide; FVC: Forced vital capacity; ICU: Intensive care unit; ILD: Interstitial lung disease; TLC: Total lung capacity

**Table 2:** Pre-existing Comorbidities

	Bern Non-ICU (n = 99)	Bern Post-ICU (n = 25)	Lausanne Post-ICU (n = 68)
**Asthma**	14 (14.1%)	1 (4.0%)	10 (14.9%)
**ILD**	1 (1.0%)	2 (8.0%)	0 (0.0%)
**COPD**	0 (0.0%)	0 (0.0%)	2 (3.0%)
**Cor. Art. Disease**	5 (5.1%)	2 (8.0%)	3 (4.5%)
**Art. Hypertension**	10 (10.2%)	11 (44.0%)	32 (47.8%)
**Heart failure**	1 (1.0%)	2 (8.3%)	5 (7.5%)
**Pulm. Embolism**	1 (1.0%)	3 (12.0%)	1 (1.5%)

Abbreviation: COPD: Chronic obstructive pulmonary disease; ICU: Intensive care unit; ILD: Interstitial lung disease

Lung function measurements performed 4 months after COVID-19 showed that patients with a previous ICU stay had reduced pulmonary function measurements compared to non-ICU patients after four months as we have previously reported [6]. Bern and Lausanne post-ICU groups had reduced diffusing capacity for carbon monoxide (DLCO = 75.96% predicted and 71.11% predicted, respectively) compared to non-ICU (p < 0.0001). In addition, predicted forced vital capacity (FVC) and total lung capacity (TLC) were also significantly lower in Bern (FVC = 87.65% predicted, TLC = 86.70% predicted) and Lausanne post-ICU (FVC = 87.11% predicted, TLC = 82.91% predicted) than in non-ICU (FVC = 97.40% predicted, TLC = 100.98% predicted) (p < 0.012, p = 0.001, respectively).

Based on their DLCO measurements, patients were subdivided in two groups: Impaired DLCO (below 80% predicted) or non-impaired DLCO ( > = 80% predicted). We observed that the incidence of DLCO impaired patients in the Bern (58.3%) and Lausanne post-ICU cohort (67.2%) was greater than in the Bern non-ICU cohort (18.4%) (p < 0.0001 each). Moreover, during six-minute walking test, post-ICU patients had a larger transcutaneous oxygen desaturation (Bern = 5.17% desaturation, Lausanne = 6.86% desaturation) than non-ICU patients (3.84%, p < 0.039).

The most frequent pre-existing comorbidity in all the three groups was arterial hypertension: > 40% of the participants in the Bern and Lausanne post-ICU group and 10.2% in Bern non-ICU group, followed by asthma: 14.1% of the participants in Bern non-ICU and 14.9% in Lausanne post-ICU.

### Serum calprotectin levels are higher in post-ICU COVID-19 patients

**Fig. 2:**
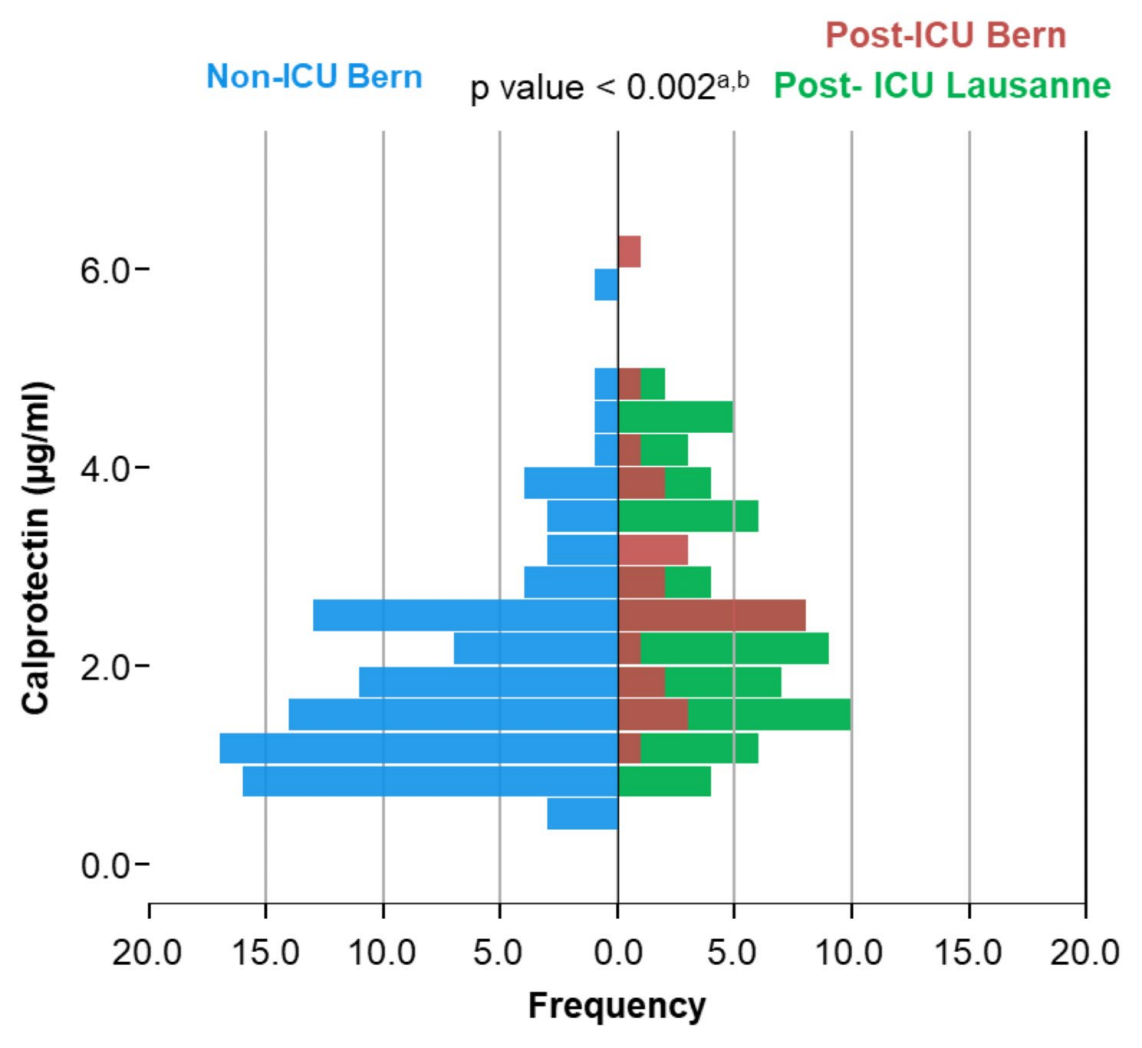
Calprotectin in Post-COVID-19: Bern and Lausanne Cohorts Frequencies of serum calprotectin levels (µg/ml) in the non-ICU cohort (blue) vs. the post-ICU cohorts (Bern: red, Lausanne: green)

Peripheral blood samples were collected at time of pulmonary function testing. Serum calprotectin measurements revealed that post-ICU patients each had significantly higher calprotectin levels (Bern = 2.74 ± 1.15 µg/ml, Lausanne = 2.49 ± 1.13 µg/ml) compared to non-ICU patients (1.86 ± 1.02 µg/ml, p < 0.002 each, Fig. 2). Meanwhile, there was no statistical difference between the two post-ICU groups.

**Fig. 3:**
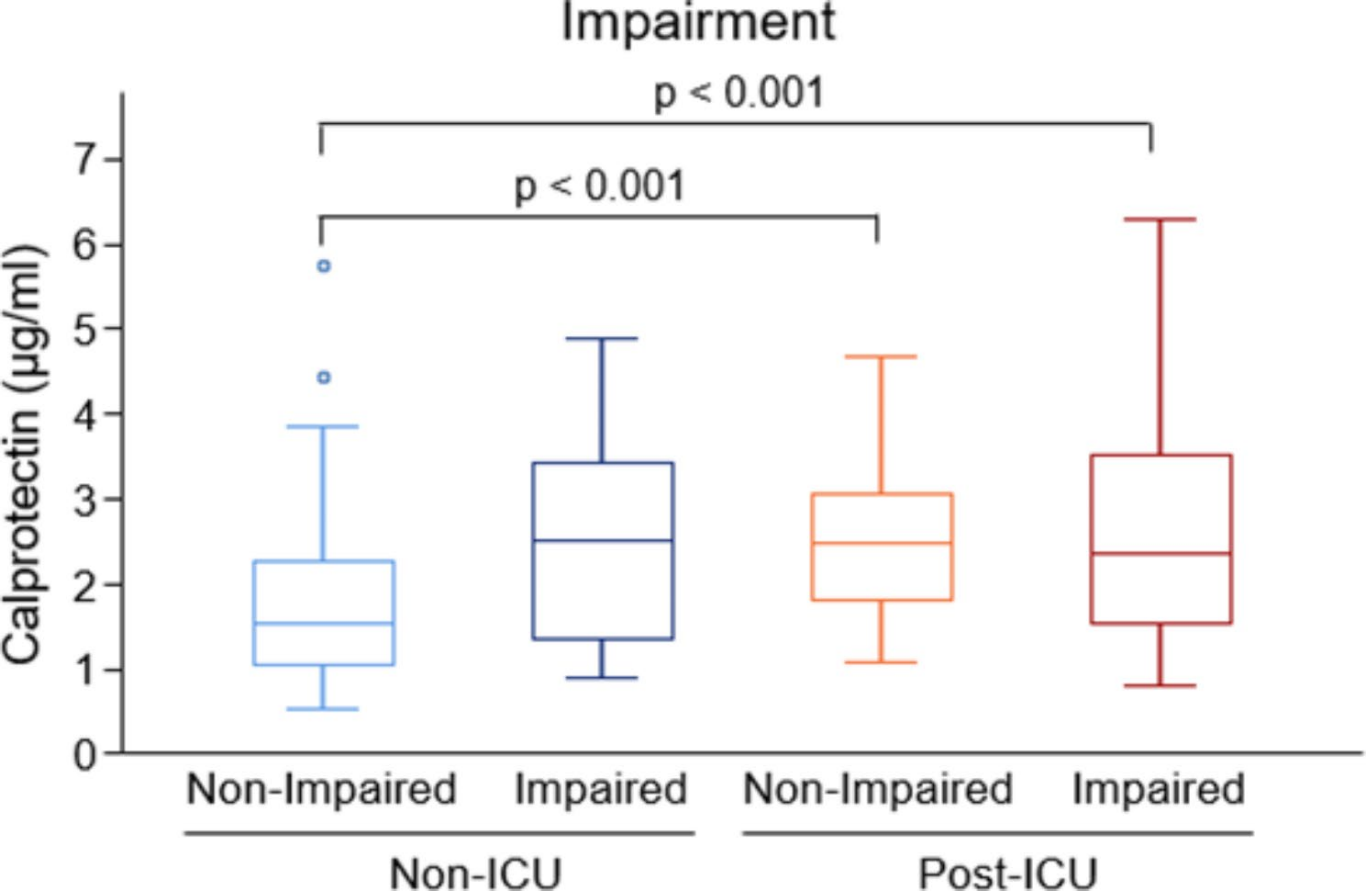
Calprotectin and Lung Impairment in Post-COVID-19 Distributions of serum calprotectin levels (µg/ml) in non-ICU patients vs. post-ICU patients according to their lung status: non-impaired (DLCO ≥ 80%) or impaired (DLCO < 80%).

Furthermore, to investigate whether serum calprotectin levels differed between patients with and without lung impairment, both cohorts were classified according to the ICU admission and the % predicted DLCO (Fig. 3). The group of non-ICU patients without impairment had the lowest serum calprotectin level (Fig. 3). On average, these patients had less serum calprotectin than the post-ICU patients with or without lung impairment (DLCO < 80% predicted). Calprotectin concentrations were similar between non-ICU patients with and without impairment four months after the infection, as well as between post-ICU patients with and without lung impairment.

A more detailed version of this comparison between impaired and non-impaired patient groups, where the post-ICU cohorts are split, is shown in the supplemental Fig. 1. In all patients combined, the comparison between impaired and non-impaired patients was significant (p = 0.002).

### Serum calprotectin correlate with lung function

**Fig. 4:**
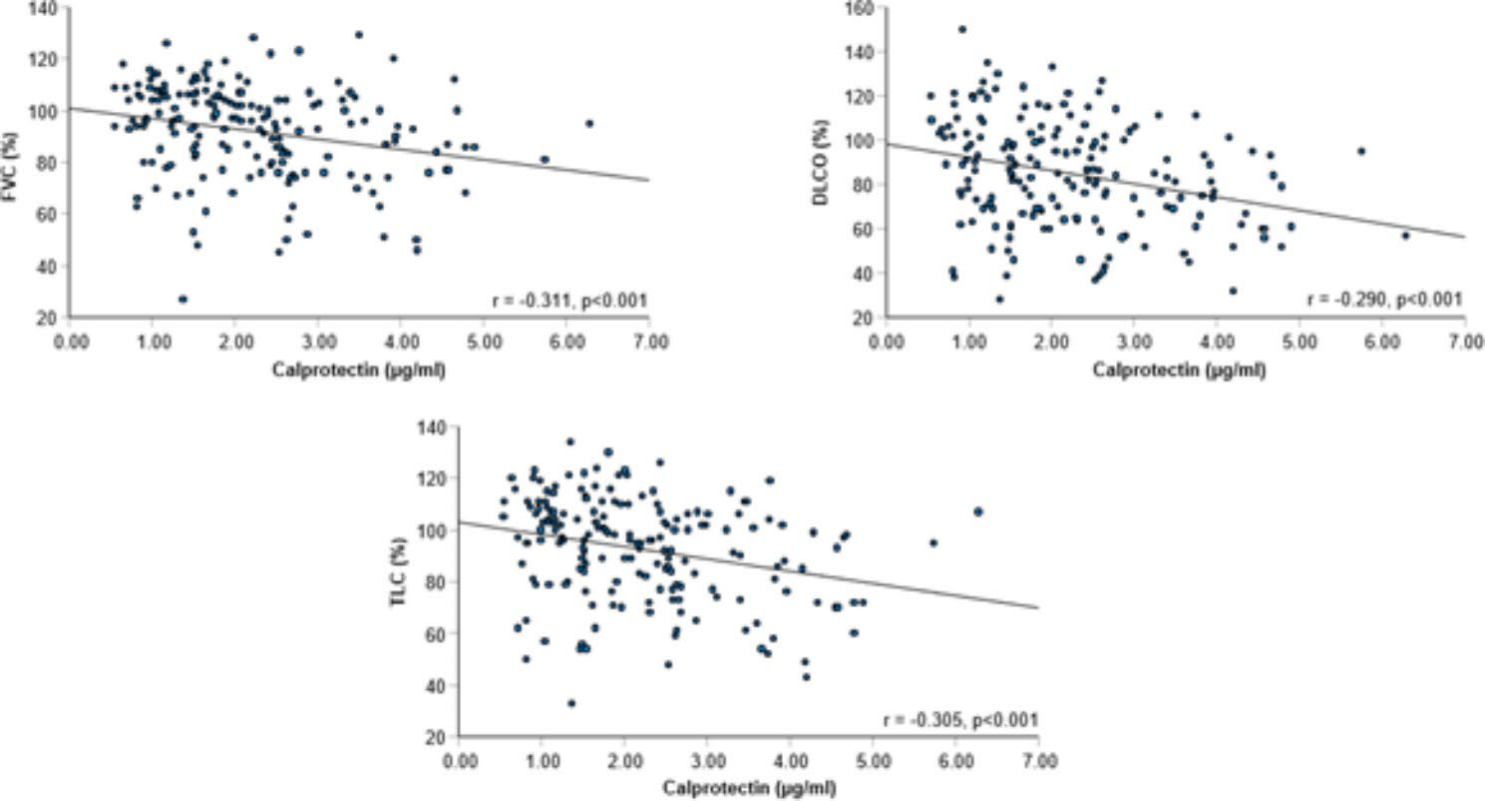
Correlation between Calprotectin and Pulmonary Function in Post-COVID-19 Correlation between serum calprotectin levels and % predicted FVC, TLC, and DLCO within the whole cohort

Serum calprotectin levels showed a weak but significant negative correlation with FVC% predicted (r = -0.311), TLC% predicted (r = -0.305), and DLCO% predicted (r = -0.290; all p < 0.001; Fig. 4).

To investigate possible dependencies of calprotectin on other factors such as sex, age, BMI, lung function 4 months after COVID-19, or comorbidities, multiple linear regression was performed. As presented in the regression model in Table 3 with calprotectin as the dependent variable, a significant association was found with age (coefficient 0.017, CI 0.005 to 0.028, p = 0.004), BMI (0.041, CI 0.015 to 0.067, p = 0.003), and desaturation during six-minute walking test (0.063, CI 0.031 to 0.094, p < 0.0001).

**Table 3:** Regression Model

Model	Coefficient	95% CI	p-value
Constant	-0.088	-0.963 to 0.788	0.843
Age	0.017	0.005 to 0.028	0.004
BMI	0.041	0.015 to 0.067	0.003
Desaturation	0.063	0.031 to 0.094	< 0.0001

r = 0.437; adjusted R^2^ = 0.178; ANOVA p-value < 0.001

## Discussion

Our results showed that serum calprotectin levels four months after acute SARS-CoV-2 infection were significantly higher in patients that had a severe course of COVID-19 and initially required ICU admission compared to patients with milder disease. These results were validated in post-ICU patients from Lausanne. To our knowledge, this is the first time that calprotectin concentrations have been studied and compared in post-COVID patients with distinct primary disease severities. Furthermore, patients that were in the ICU had reduced lung function with significantly lower DLCO compared to patients without ICU admission.

The lung functional impairment coincides well with other studies that described reduced lung diffusion capacity after severe COVID-19 compared to mild/moderate disease courses [28, 29]. The reason for the DLCO impairment observed in some patients post COVID-19 are not fully understood, but there is evidence that air trapping due to small-airway inflammation may be involved. We have observed mosaic pattern as a radiological sign for air trapping in chest scans 12 months after infection [7]. Another study has described air trapping as a common finding in expiratory CT scans of post-COVID patients with persisting respiratory symptoms [30]. An association of air trapping and reduced DLCO has also been described [31]. Furthermore, some studies have described small airway disease and the mismatch between ventilation and perfusion using VQ SPECT/CT as another cause for diffusion capacity impairment after COVID-19 [30]. Also, ongoing inflammation is among the most debated hypotheses for long COVID [15], that might lead to obstruction of small vessels and/or airways leading to our observations.

Within our entire cohort calprotectin levels were negatively correlated with pulmonary function.

These findings confirm that inflammation and lung impairment persist after severe COVID-19. Moreover, they suggest that calprotectin or at least ongoing inflammation may be involved as a mechanism of persisting lung impairment after COVID-19.

Literature on calprotectin in post-COVID patients is limited to a few publications that investigated a group of convalescent blood donors [32] and a population up to 6 weeks after mild COVID-19 [33]. Calprotectin is a promising biomarker for various diseases as it reflects inflammatory activity [23–26]. It is straightforward to measure in clinical routine. In COVID-19, several studies have already described that calprotectin levels correlated with the severity of the acute disease [18–22]. Circulating calprotectin differentiated between patients with severe, moderate, and mild COVID-19, respectively [18]. Moreover, calprotectin was predictive for disease severity, ICU admission, intubation, and death in patients with acute COVID-19 [19–22]. Currently, research groups seek biomarkers to monitor long COVID patients [34–36], hence a biomarker such as Calprotectin for persistent disease activity that can easily be measured could assist clinicians to manage the disease effects post COVID-19.

According to the current literature, knowledge about calprotectin in post-COVID-19 patients is scarce. Nevertheless, there are a few publications that indicate that calprotectin may play a role in the pathophysiology of persisting symptoms after acute COVID-19. One study performed consecutive plasma proteome analysis from 1 to 6 weeks after mild SARS-CoV-2 infection in healthcare workers and found a trend for higher serum calprotectin levels at the time of seroconversion in individuals that developed persistent symptoms. In that study, calprotectin was one of the biomarkers with the strongest association with long-term symptoms captured in questionnaires 6 and 12 months after COVID-19 [33].

Another study looked for 102 different autoantibodies in the plasma of convalescent healthcare workers 8 months after their acute SARS-CoV-2 infection as well as in uninfected controls. Here, anti-calprotectin autoantibodies appeared to be protective. With a positivity rate of 22.6% in the convalescent group, anti-calprotectin autoantibodies were the most frequently detected autoantibodies and individuals with positive titres for anti-calprotectin autoantibodies were significantly more likely to report full clinical recovery 8 months post-COVID-19 compared to individuals without these autoantibodies [37].

A recent study showed a persistent neutrophil-associated immune signature in patients with post-COVID pulmonary sequelae [38]. As calprotectin is produced by neutrophils, our findings may align with this recent publication and offer an available surrogate clinical test for neutrophil inflammation.

Our study has several limitations. Firstly, most of our patients have lung function impairment, as most of the patients with no respiratory symptoms were excluded from the original study, and in the Lausanne part of the cohort only severe COVID cases post-ICU were included in the cohort which may lead to selection bias. Furthermore, only few of the patients in both cohorts had previously known relevant lung disease such as ILD and we did not have any lung function data before COVID-19 infection. By this relatively small sample size and limited number of comorbidities a further subgroup analysis was not feasible. In addition, serum calprotectin was not determined at the time of initial hospital admission. For this reason, no statement can be made about the trends of caprotectin levels over time. For example, it remains unclear whether those patients who still had high calprotection levels after 4 months already had high levels at the onset of the disease. Finally, the fact that it is an observational cohort study we cannot claim causality, thus more studies are needed to investigate pathways and our findings need to be verified in a larger context of long COVID research.

## Conclusions

Four months after acute COVID-19, serum calprotectin concentrations were significantly higher in patients that had a severe disease course with ICU admission compared to mildly/moderately affected patients. In addition, calprotectin and DLCO correlate negatively. This suggests an involvement of continual inflammation and calprotectin in the pathophysiology of persisting lung impairment after COVID-19. Further research is needed to better understand the association between inflammation and persisting pulmonary dysfunction after COVID-19 and to determine whether calprotectin could be a candidate biomarker to monitor long-COVID patients.

### Electronic supplementary material

Below is the link to the electronic supplementary material.

Supplementary Material 1

## Data Availability

The datasets used and/or analysed during the current study are available from the corresponding author on reasonable request.

## Abbreviations

ARDS: Acute Respiratory Distress Syndrome
BMI: Body Mass Index
COVID-19: Coronavirus Disease 2019
DLCO: Diffusion Capacity of the Lungs for Carbon Monoxide (CO)
FVC: Forced Vital Capacity
ICU: Intensive Care Unit
IPF: Idiopathic Pulmonary Fibrosis
MERS: Middle East Respiratory Syndrome
SARS: Severe Acute Respiratory Syndrome
TLC: Total Lung Capacity
