# Supplementary material for: Circulating calprotectin levels four months after severe and non-severe COVID-19
Source: BMC Infect Dis. 2023 Oct 3;23:650. doi: 10.1186/s12879-023-08653-7 (PMC10546778; doi:10.1186/s12879-023-08653-7)
Supplement: Supplementary file 1 — Supplementary Material 1 [file 12879_2023_8653_MOESM1_ESM.docx]

**Supplemental Figure 1: Calprotectin and Lung Impairment in Post-COVID-19**


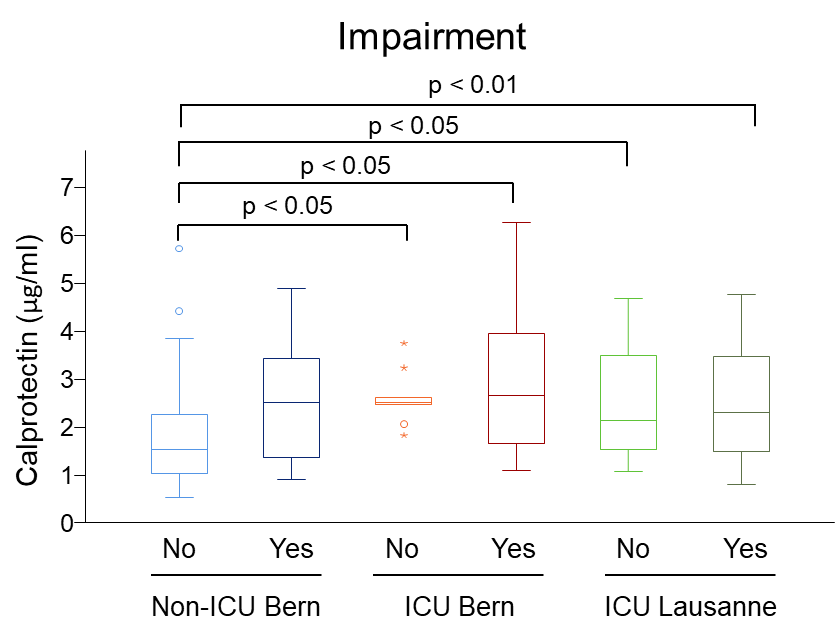


Distributions of serum calprotectin levels (μg/ml) of all three cohorts differentiated in non-impaired (DLCO ≥ 80%) and impaired (DLCO < 80%) patient groups.
